# Changes in continuum beliefs for depression and schizophrenia in the general population 2011–2020: a widening gap

**DOI:** 10.1007/s00127-022-02272-4

**Published:** 2022-03-19

**Authors:** Georg Schomerus, Stephanie Schindler, Eva Baumann, Matthias C. Angermeyer

**Affiliations:** 1 Department of Psychiatry, University of Leipzig Medical Center, Semmelweisstr. 10, 04103 Leipzig, Germany; 2 Department of Journalism and Communication Research, Hannover University of Music, Drama, and Media, Hannover, Germany; 3 Center for Public Mental Health, Gösing am Wagram, Austria

**Keywords:** Stigma, Continuum beliefs, Attitudes, Population survey

## Abstract

**Purpose:**

The public discourse about mental health and mental illness seems to have become more open over the last decade, giving rise to the hope that symptoms of mental illness have become more relatable. We examine whether continuum beliefs regarding schizophrenia and depression have increased on a population level over a period of 9 years, and whether notions of unfamiliarity and incomprehensibility have decreased.

**Methods:**

In 2011 (*n* = 2455) and 2020 (*n* = 3042), two methodologically identical cross-sectional population surveys were conducted in Germany. After the presentation of an unlabeled case vignette depicting someone with schizophrenia or depression, we asked about continuum beliefs, perceived unfamiliarity and perceived incomprehensibility of the person described.

**Results:**

Multinomial logit models holding sociodemographic variables fixed at their means for both surveys showed that agreement with continuum beliefs increased in depression from 43 to 46% [change 4%; 95% confidence interval (CI) 0; 8], but decreased in schizophrenia from 26 to 20% (change − 6%, 95% CI − 9; − 3). Unfamiliarity (change − 4%; 95% CI − 7; 0) and incomprehensibility (change − 7%, 95% CI − 10; − 4) decreased for depression, while remaining largely unchanged for schizophrenia.

**Conclusion:**

An already pronounced gap in the perception of both disorders with regard to continuity of symptom experiences and perceived otherness further widened over the last decade. While the public’s increasing familiarity with symptoms of depression might be further aided by using continuum beliefs as part of anti-stigma messages and awareness campaigns, promulgating continuity models for schizophrenia seems more challenging.

**Supplementary Information:**

The online version contains supplementary material available at 10.1007/s00127-022-02272-4.

## Introduction

Over the last decade, the public discourse about mental illness has become more open. Several celebrities have come out with their own mental illness [14]. Highly visible anti-stigma and public awareness campaigns like Time to Change, or the European Alliance Against Depression, have kept mental health issues in the news, often with the help of well-known patrons. In many contexts, talking about one’s own mental health problems seems to have become easier and increasingly normal [25]. In Germany, epidemiological studies show that while the prevalence of mental disorders like depression is overall stable [5], sick leaves and disability pensions due to mental health problems have increased [7, 8]. Taken together, it seems as if, at least for a proportion of the general public, mental illness has become a more familiar topic that is now more relatable to personal experiences than it was previously.

Relating personal experiences to possible mental illness, or acknowledging an occasional similarity of own experiences with the experiences of people with mental illness, could be seen as indicating an individual’s belief that symptoms of mental illness are on a continuum with mental health. A continuum model of mental health and illness maintains that differences between mental illness and mental health are gradual rather than categorical, and that most people at some point experience symptoms similar to those during an episode of mental illness. Continuum beliefs have been shown to be related to less stigmatizing attitudes [2, 22, 27, 28, 30, 31]. A recent systematic review and meta-analysis found that continuum beliefs are associated with lower social distance, lower fear, lower perceptions of dangerousness and unpredictability, and stronger pro-social reactions [16]. The review also demonstrated that continuum beliefs differ between disorders, they are for example generally higher with regard to depression, and lower with regard to schizophrenia. Overall, continuum beliefs are regarded as countering the perceived “otherness” of someone with mental illness, because they provide a connection of perceived similarity between “us” and people with mental health problems [16].

Given the growing openness surrounding mental health problems over the last years, the question arises whether continuum beliefs have increased on a population level. If symptoms of mental illness have indeed become more relatable to members of the general public, continuum beliefs should also have increased. So far, no time-trend studies on the evolution of continuum beliefs among the general public exist. Using data from two methodologically identical population surveys in Germany conducted in 2011 and 2020, we examine whether the prevalence of continuum beliefs has changed over the last decade. Additionally, we look at changes in the notion of being different, and of being incomprehensible, as further attributes illustrating the perceived “otherness” of someone with mental illness. Surveys were based on unlabeled case vignettes depicting someone with either schizophrenia or depression. We hypothesize that continuum beliefs related to both disorders have increased and that the perceived “otherness” of someone with mental illness has decreased.

## Methods

### Sampling strategy

The two population surveys were conducted face-to-face in 2011 (*n* = 2455, response rate 64%) and 2020 (*n* = 3042, response rate 57%) among people living in Germany, aged 18 years and older. Both surveys used identical sampling and interview methodology to enable time trend analyses. Samples were drawn using a random sampling procedure with three stages: (a) sample points (electoral wards), (b) households, and (c) individuals within target households. Target households within sample points were determined according to the random route procedure, that is, a street was selected randomly as a starting point from which interviewers followed a set route through the area. Target individuals were selected using random digits. Informed consent was considered to have been given when individuals agreed to complete the interview. Fieldwork for both surveys was carried out by USUMA (Berlin), a company specialized in market and social research.

### Sample

Table 1 shows sociodemographic characteristics of the two samples and the general population at the time of the surveys. Except for education, where highly educated people were under-represented in 2011 and 2020, our samples can be considered representative of the German population.

**Table 1:** Socio-demographic characteristics of the population samples

	2011		2020	
	Survey	Total population	Survey	Total population
Gender				
Male	45.6	48.6	47.2	48.9
Female	54.4	51.4	52.4	51.1
Diverse	n.a	n.a	0.4	n.a
Age, years				
18–25	8.4	11.3	10.4	10.4
26–45	29.8	31.9	32.3	29.9
46–60	28.5	26.9	28.5	27.3
61 +	33.3	29.9	28.9	32.4
Educational attainment				
Still student	0.8	1	0.9	0.6
No schooling completed	3.3	4	1.6	4.1
8/9 years of schooling	38.6	38.5	28.2	29.8
10 years of schooling	39.1	29.3	41.2	30.7
12/13 years of schooling	18.2	27.1	28.1	34.8

Percentages of sample/population. Population Data from the Federal Statistical Office of Germany

*n.a.* not available

### Interview

Both surveys were carried out as in person, face-to-face interviews by trained interviewers using paper and pencil. The fully structured interviews were identical regarding the interview methodology, structure, and wording of questions and reply options for all measures used in this analysis. Interviews started by presenting a diagnostically unlabeled psychiatric case history (vignette). Respondents were randomly assigned either a description of someone with schizophrenia or major depressive disorder. The symptoms described fulfilled the criteria of DSM-III-R for the respective disorder and were first used in a population survey in 1990 [1]. Before being used, both vignettes had been rated by five experts in psychopathology, confirming the correct diagnosis. The gender of the vignette varied at random (see online supplement for the wording of the vignettes) The depression vignette was presented to *n* = 1220 respondents in 2011, and *n* = 1530 in 2020; the schizophrenia vignette was presented to *n* = 1235 in 2011 and *n* = 1512 in 2020. We asked respondents about a wide range of attitudes and beliefs both with regard to the person described and unrelated to the case vignette. For this study, the following measures were used:

### Measures

After the presentation of the vignette, we asked respondents to what extent they believed that the person described was “unfamiliar” or “incomprehensible”. For both attributes, answers had to be given on a five-point Likert-scale, ‘‘1’’ indicating strong agreement and ‘‘5’’ indicating strong disagreement with the statement.

To measure continuum beliefs, we asked respondents to indicate their agreement with the following statement, that has first been used to assess continuum beliefs in 2011 [22] and has since been employed in various studies [2, 26, 27]: ‘‘Basically we are all sometimes like this person. It’s just a question of how pronounced this state is.’’ Again, we recorded answers on a five-point Likert-scale, ‘‘1’’ indicating strong agreement and ‘‘5’’ indicating strong disagreement.

We further elicited the respondents’ gender, years of schooling completed, and age.

### Statistical analysis

To ease interpretation of changes over time and to counterbalance tendencies to preferably select or avoid the extreme response categories, we inverted the ratings for each item and combined the two values below and above the midpoint, resulting in three categories 1 = “disagree”, 2 = “undecided” and 3 = “agree”.

For each item, we calculated a multinomial logistic regression with time, vignette, the interaction of time and vignette, gender of the vignette as well as age, gender, and education of the respondents as predictors. To ensure the stability of the model estimation we excluded subjects with diverse gender and collapsed educational attainment into three categories (< 10 years, 10 years, and > 10 years of schooling completed). We then used the logistic models to predict the probability of choosing a particular response category irrespective of potential demographic changes between the two samples from the years 2011 and 2020. To estimate the changes in answer behavior from 2011 to 2020 we calculated the average change in predicted probability for each response category separately for each vignette while holding all control variables constant at their means of the full dataset. It serves as an unstandardized effect size of the change. All probabilities and discrete changes were multiplied by 100 and can thus be read as (change in) percentages of respondents choosing each answer category. The delta method was used to compute 95% confidence intervals [CI].

## Results

Pairwise correlation analysis of continuum beliefs, and perceptions of unfamiliarity and incomprehensibility showed moderate intercorrelations between unfamiliarity and incomprehensibility (*r* = 0.52 in schizophrenia; *r* = 0.57 in depression), low negative correlations between continuum belief and unfamiliarity (schizophrenia, *r* = − 0.22; depression, *r* = − 0.16), and low negative correlations between continuum beliefs and incomprehensibility (schizophrenia, *r* = − 0.14; depression, *r* = − 0.16, all pairwise correlations *p* < 0.001), illustrating that our items captured distinct, but interrelated aspects of “otherness”.

Table 2 shows the predicted probabilities for the three answer categories for all three dependent variables examined. Probabilities can be read as percentages of the population endorsing the respective answer, adjusted for the gender of the vignette, as well as age, gender, and education of the respondents. Generally, agreement with continuum beliefs was about twice as high for depression compared to schizophrenia (Table 2).

**Table 2:** Changes in predicted probabilities for endorsing continuum beliefs, unfamiliarity, and incomprehensibility for depression or schizophrenia

Statement (*N*)	Response category	Depression				Schizophrenia			
		2011	2020	Change	95% CI	2011	2020	Change	95% CI
Continuum belief (*n* = 5416)	Agree	**42.5**	**46.3**	3.9*	**0.1, 7.7**	**26.1**	**19.8**	− 6.2***	− **9.5, **− **3.0**
	Undecided	**32.7**	**28.8**	− 4.0*	− **7.5, **− **0.4**	24.6	25.3	0.7	− 2.6, 4.0
	Disagree	24.8	24.9	0.1	− 3.2, 3.4	**49.4**	**54.9**	5.5***	**1.7, 9.3**
Unfamiliar (*n* = 5405)	Agree	**26.7**	**23.2**	− 3.5*	− **6.8, **− **0.2**	51.9	48.4	− 3.6	− 7.4, 0.3
	Undecided	**29.2**	**23.1**	− 6.1***	− **9.4, **− **2.7**	24.8	27.8	2.9	− 0.4, 6.3
	Disagree	**44.1**	**53.8**	9.6***	**5.8, 13.4**	23.2	23.9	0.6	− 2.6, 3.9
Incomprehensible (*n* = 5399)	Agree	**27.0**	**20.3**	− 6.8***	− **10.0, **− **3.5**	38.3	38.1	− 0.2	− 3.9, 3.5
	Undecided	25.7	23.1	− 2.6	− 5.9, 0.7	**29.1**	**25.0**	− 4.1*	− **7.5, **− **0.7**
	Disagree	**47.3**	**56.7**	9.4***	**5.5, 13.2**	**32.6**	**36.9**	4.3*	**0.6, 7.9**

Percentages of response categories and their discrete change from 2011 to 2020 were predicted using a multinomial logistic regression with a period, vignette, interaction period*vignette, and gender of the vignette, as well as age, gender, and educational attainment of the respondent as predictors. All covariates were evaluated at their means of the combined sample. Statistically significant differences are highlighted in bold font. *N* sample size

*p < 0.05, ***p < 0.001

As expected, continuum beliefs regarding depression gained in popularity between 2011 and 2020. While 43% agreed with a continuum statement in 2011, 46% agreed in 2020 (change in predicted probability, 4%). Conversely, notions of unfamiliarity became less prevalent: agreement decreased from 27% in 2011 to 23% in 2020, disagreement increased from 44 to 54%. Similarly, perceiving the person with depression as incomprehensible decreased from 27 to 20% (disagreement with this statement increased from 47 to 57%).

In schizophrenia, in contrast, we saw a further reduction in agreement with continuum beliefs. While 26% had agreed with continuum beliefs in 2011, this share fell to 20% in 2020, a decrease of 6%. Rejection of continuum beliefs increased, from 49% in 2011 to 55% in 2020. Also, the marked decrease of perceived “otherness” seen for depression was almost absent in schizophrenia, where ratings remained comparatively high at about 50% regarding unfamiliarity, and at about 38% regarding incomprehensibility. Only disagreement with seeing the person as incomprehensible increased from 33 to 37% (Table 2).

Results for the full multinomial logit models are provided in supplementary table 1. Among the co-variates, higher education was generally associated with more agreement with continuum beliefs, and more disagreement with notions of incomprehensibility. Female participants were also more likely to disagree with the person being incomprehensible.

As a sensitivity analysis, we calculated linear regression models, treating the five-point outcome variables as continuous variables and using identical predictor variables. Similar to our multinomial logit models, this yielded a significant increase in continuum beliefs in depression, a decrease in schizophrenia, and decreasing unfamiliarity and incomprehensibility in depression (see supplementary table S2).

## Discussion

Summarizing our findings, we did not find a general increase of continuum beliefs regarding mental illness, but rather a distinct development for depression on the one side, and schizophrenia on the other. Regarding depression, our study revealed an increase in continuum beliefs, and a marked decrease in perceived “otherness” over the last decade. In contrast, continuum beliefs with regard to schizophrenia became less popular, while perceptions of unfamiliarity and incomprehensibility remained largely unchanged. We thus observed a widening gap between attitudes towards someone with depression and schizophrenia with regard to continuum beliefs and perceived incomprehensibility and unfamiliarity. While the symptoms of depression have become even more relatable over the last decade, symptoms of schizophrenia are seen even less on a continuum with the respondents own experiences than they were 9 years ago.

Before discussing our findings in detail, we look at the limitations of our study. First, continuum beliefs and perceived otherness were measured with single items, which could impair the validity of our findings. Our item on continuum beliefs has meanwhile been used in several studies, including experimental studies [6, 21], where it has been used in combination with other items and has shown to validly assess continuum beliefs. Furthermore, correlation analysis showed the expected interrelations between variables. Second, since this is an observational time trend study, no conclusions on causality for the observed developments can be made. However, since results differ for depression and schizophrenia, there is a strong argument that developments are specific for the type of illness described, and not due to some general trend unrelated to mental illness. Thirdly, since both surveys were conducted 9 years apart, the question arises whether our findings represent a sustained trend or are the result of random volatility of attitudes. Other studies have shown very little volatility of population attitudes regarding mental illness. Repeated surveys before and after the Germanwings plane crash in Germany, for example, showed only a few and small changes, consistent with the anticipated increases in notions of dangerousness and unpredictability [11, 23]. Annual surveys in England showed steady changes consistent with the messages of the Time to Change campaign, also with little volatility [18]. Furthermore, as we will discuss below, our findings correspond to results of a time-trend study from the US, which also found a widening gap between attitudes toward schizophrenia and depression [15], and to changes in resource allocation preferences of the public in Germany [21]. A particular strength of our study is the use of identical case vignettes in two large representative surveys using identical sampling and interview methodology. By using descriptions of symptoms and behavior, changes in attitudes are clearly related to the depicted mental illness, and not subject to changing significances of labels like “mental health problems”, that have been used in other time trend studies [18].

Our finding that continuum beliefs are increasing with regard to someone with depression corroborates the notion that public discourse about depressive disorders is becoming more open and familiar. For example, while 36% of respondents in an Austrian population survey in 2007 stated that they knew someone with depression in their family or among their close friends, these numbers had risen to 50% in 2018 [10], indicating that more and more people come out with their depressive disorder.

Another indicator that the symptoms of depression are increasingly perceived as being closer to peoples everyday experiences is that depression is most prevalent in media coverage. A recent content analysis of three German newspapers revealed a clear discrepancy in media reports on different types of mental disorders with most articles referring to depression (19%), while schizophrenia and other psychotic disorders were subject of reporting in only 10% [19]. Further, there is a growing popularity of “burn-out” in public discourse. Another media analysis in Germany showed that symptoms reported in connection with “burn-out” widely overlap with those of depression [17]. In fact, the number of respondents using the label “burn-out” when presented with a vignette showing the symptoms of depression grew from 0.3% in 2001 to 10.2% in 2011 [4]. Symptoms of depression seem to fit into a popular notion of being overwhelmed, overburdened and stressed by the demands of accelerating modern life [9].

Our finding that the symptoms of depression have become more relatable and less incomprehensible for the general public aligns with a growing readiness of the German public to prioritize funding for depression treatment. Over the course of 20 years, funding healthcare for depression rose from a second-last position among nine mental and medical disorders to the fourth position, prioritized by one in four respondents [21]. This is a welcome development and likely makes coming out with depression and seeking help easier. Our findings also align with a trend study from the U.S. that found reduced social distance towards someone with depression (but not with schizophrenia) particularly between 2006 and 2018. In the most recent U.S. survey, the level of rejection of someone with depression resembled the rejection of someone with daily troubles [15].

However, the growing familiarity with symptoms of depression does not sufficiently explain the entirety of our findings, because it does not account for the negative changes we found with regard to schizophrenia. While the behavior described in the schizophrenia vignette could have been expected to elicit more skeptical reactions than the depression vignette, we do not have an explanation why beliefs in a continuity of symptoms of schizophrenia have decreased since 2011. Obviously, research demonstrating how common psychosis-like symptoms are among the general population [13], and the debate about the continuum of psychotic experiences [29], do not seem to have found their way into the public discourse.

One possible explanation could be that the media frame mental illnesses quite differently. Depression is comparatively often framed as a health problem, but reports on schizophrenia only sparsely refer to symptoms and treatment options, but more on violent incidents caused by people with psychotic illness [3, 19]. However, there have also been reports on violent incidents related to depression: a major flight disaster, the crash of a passenger plane intentionally brought down by its pilot, was explicitly linked to a diagnosis of depression and widely covered by German Media in 2015. Against all expectations, this incident did not meaningfully worsen depression or mental illness stigma, as was shown by two independent population studies [11, 23]. Similarly, a series of violent attacks in 2016 that had been linked (among other things) to mental illness, did not cause immediate shifts in attitudes towards people with mental illness [24]. So it seems unlikely that media reporting alone caused this divergent time trend for both disorders. We can only speculate whether broad societal trends like an increasing desire for security and controllability in everyday life contribute to the growing unfamiliarity of someone with schizophrenia [20].

Regardless of its causes, the current development is worrying for people with severe psychotic illnesses-like schizophrenia. Perceived “otherness” is at the core of the stigma process as conceptualized by Link and Phelan [12], and although the overall increases in “otherness” (as indicated by lower continuum beliefs) of someone with schizophrenia seen in this study are small, we consider them meaningful. From the initially high levels of otherness in schizophrenia, and low levels in depression, a “regression to the mean” would have yielded the exact opposite development than what was observed. Together with the above-cited findings on changing resource allocation preferences, and the divergent development of attitudes towards schizophrenia and depression observed in the U.S., our findings underline that de-stigmatizing depression does not automatically decrease stigma for other mental disorders. There seems, at least so far, no “trickle down” of de-stigmatization of common mental disorders to those experiencing severe, psychotic illness.

There is preliminary evidence that continuum messages can be used to reduce the stigma of mental illness [16, 30]. If continuum beliefs are to be used as a strategy against stigma, our findings show that they are likely to be well received with regard to depression, fueling a trend that has already materialized on a population level. Promulgating a continuum concept of psychosis, in contrast, is likely to be more challenging since this would run counter the time trend observed in this study. Given the persistence of stigma particularly with regard to severe mental illness, however, this effort could well be worthwhile.

## Supplementary Information

Below is the link to the electronic supplementary material.

Supplementary file1 (DOCX 30 kb)

## Data Availability

All data reported in this article is available from the first author upon request.

## Code availability

The Stata code used for the analyses reported in this paper is available from the first author upon request.

## References

[1] Angermeyer MC, Matschinger H (1997). Social distance towards the mentally ill: results of representative surveys in the federal republic of Germany. Psychol Med.

[2] Angermeyer MC, Millier A, Rémuzat C, Refaï T, Schomerus G, Toumi M (2015). Continuum beliefs and attitudes towards people with mental illness: results from a national survey in France. Int J Soc Psychiatry.

[3] Angermeyer MC, Schulze B (2001). Reinforcing stereotypes: How the focus on forensic cases in news reporting May influence public attitudes towards the mentally ill. Int J Law Psychiatry.

[4] Bahlmann J, Angermeyer MC, Schomerus G (2013). "Burnout" statt "Depression"—eine Strategie zur Vermeidung von Stigma?. Psychiatr Prax.

[5] Bretschneider J, Janitza S, Jacobi F, Thom J, Hapke U, Kurth T, Maske UE (2018). Time trends in depression prevalence and health-related correlates: results from population-based surveys in Germany 1997–1999 vs. 2009–2012. BMC Psychiatry.

[6] Buckwitz V, Porter PA, Bommes JN, Schomerus G, Hinshaw SP (2021). Continuum beliefs and the stigma of depression: an online investigation. Stigma Health.

[7] DAK (2013) DAK-Gesundheitsreport 2013. https://www.dak.de/dak/download/vollstaendiger-bundesweiter-gesundheitsreport-2013-2120160.pdf. Accessed 1 Feb 2022

[8] Deutsche Rentenversicherung Bund (2020) Rentenversicherung in Zeitreihen: DRV-Schriften-Band 22. https://www.deutsche-rentenversicherung.de/SharedDocs/Downloads/DE/Statistiken-und-Berichte/statistikpublikationen/rv_in_zeitreihen.pdf?__blob=publicationFile&v=5. Accessed 1 Feb 2022

[9] Fuchs T, Iwer L, Micali S (eds) (2018) Suhrkamp Taschenbuch Wissenschaft: vol 2252. Das überforderte Subjekt: Zeitdiagnosen einer beschleunigten Gesellschaft (Erste Auflage, Originalausgabe). Suhrkamp

[10] Grausgruber A, Moosbrugger R, Hackl E (2018) Monitoring public stigma in Austria 1998–2018. Research Report. Johannes Kepler University

[11] von dem Knesebeck O, Mnich E, Angermeyer MC, Kofahl C, Makowski A (2015). Changes in depression stigma after the germanwings crash—findings from German population surveys. J Affect Disord.

[12] Link BG, Phelan JC (2001). Conceptualizing stigma. Ann. Rev Sociol.

[13] Nuevo R, Chatterji S, Verdes E, Naidoo N, Arango C, Ayuso-Mateos JL (2012). The continuum of psychotic symptoms in the general population: a cross-national study. Schizophr Bull.

[14] Park S, Hoffner CA (2020). Tweeting about mental health to honor carrie fisher: how #inhonorofcarrie reinforced the social influence of celebrity advocacy. Comput Hum Behav.

[15] Pescosolido BA, Manago B, Monahan J (2019). Evolving public views on the likelihood of violence from people with mental illness: stigma and its consequences. Health Aff.

[16] Peter L-J, Schindler S, Sander C, Schmidt S, Muehlan H, McLaren T, Tomczyk S, Speerforck S, Schomerus G (2021). Continuum beliefs and mental illness stigma: a systematic review and meta-analysis of correlation and intervention studies. Psychol Med.

[17] Rechenberg T, Nowikow J, Schomerus G (2020). Man gesteht sich ja heutzutage keine depression mehr ein, sondern nennt es Burnout. Psychiatr Prax.

[18] Robinson EJ, Henderson C (2019). Public knowledge, attitudes, social distance and reporting contact with people with mental illness 2009–2017. Psychol Med.

[19] Rosset M, Freytag A, Dittrich A, Jaspersen M, Baumann E (2020). Psychische Erkrankungen in Medienberichten. Befunde zur Darstellung und Wahrnehmung. ComSoc Commun Soc.

[20] Schomerus G, Angermeyer MC, Vogel D, Wade NG (2022). Time trends in public stigma. The Cambridge handbook of stigma and mental health.

[21] Schomerus G, Baumann E, Sander C, Speerforck S, Angermeyer MC (2021). Some good news for psychiatry: resource allocation preferences of the public during the COVID-19 pandemic. World Psychiatry.

[22] Schomerus G, Matschinger H, Angermeyer MC (2013). Continuum beliefs and stigmatizing attitudes towards persons with schizophrenia, depression and alcohol dependence. Psychiatry Res.

[23] Schomerus G, Stolzenburg S, Angermeyer MC (2015). Impact of the germanwings plane crash on mental illness stigma: results from two population surveys in Germany before and after the incident. World Psychiatry.

[24] Schomerus G, Stolzenburg S, Bauch A, Speerforck S, Janowitz D, Angermeyer MC (2017). Shifting blame? Impact of reports of violence and mental illness in the context of terrorism on population attitudes towards persons with mental illness in Germany. Psychiatry Res.

[25] Shorter E (2013) How everyone became depressed: the rise and fall of the nervous breakdown. Oxford University Press. http://search.ebscohost.com/login.aspx?direct=true&scope=site&db=nlebk&db=nlabk&AN=678216. Accessed 1 Feb 2022

[26] Speerforck S, Stolzenburg S, Hertel J, Grabe HJ, Strauß M, Carta MG, Angermeyer MC, Schomerus G (2019). Adhd, stigma and continuum beliefs: a population survey on public attitudes towards children and adults with attention deficit hyperactivity disorder. Psychiatry Res.

[27] Subramaniam M, Abdin E, Picco L, Shahwan S, Jeyagurunathan A, Vaingankar JA, Chong SA (2017). Continuum beliefs and stigmatising beliefs about mental illness: results from an asian community survey. BMJ Open.

[28] Thibodeau R, Shanks LN, Smith BP (2018). Do continuum beliefs reduce schizophrenia stigma? Effects of a laboratory intervention on behavioral and self-reported stigma. J Behav Ther Exp Psychiatry.

[29] van Os J, Linscott RJ, Myin-Germeys I, Delespaul P, Krabbendam L (2009). A systematic review and meta-analysis of the psychosis continuum: evidence for a psychosis proneness-persistence-impairment model of psychotic disorder. Psychol Med.

[30] Violeau L, Valery K-M, Fournier T, Prouteau A (2020). How continuum beliefs can reduce stigma of schizophrenia: the role of perceived similarities. Schizophr Res.

[31] Wiesjahn M, Brabban A, Jung E, Gebauer UB, Lincoln TM (2014). Are continuum beliefs about psychotic symptoms associated with stereotypes about schizophrenia?. Psychosis.

