# Supplementary material for: Changes in continuum beliefs for depression and schizophrenia in the general population 2011–2020: a widening gap
Source: Soc Psychiatry Psychiatr Epidemiol. 2022 Mar 19;58(1):17–23. doi: 10.1007/s00127-022-02272-4 (PMC9845162; doi:10.1007/s00127-022-02272-4)
Supplement: Supplementary file 1 — Supplementary file1 (DOCX 30 kb) [file 127_2022_2272_MOESM1_ESM.docx]

**Time trends in continuum beliefs for depression and schizophrenia in the general population 2011-2020: A widening gap**

## Supplement

**Case-vignettes (she)**

**Schizophrenia**

Please imagine that you find out the following about an acquaintance with whom you are occasionally doing something in your free time:

Within the past six months, your acquaintance appears to have changed. More and more, she retreated from her friends and colleagues, up to the point of avoiding them. If someone managed to involve her in a conversation, she would address only one single topic: the question as to whether some people had the natural gift of reading other people’s thoughts. This question became her sole concern. In contrast with her previous habits, she stopped taking care of her appearance and looked increasingly untidy. At work, she seemed absent-minded and frequently made mistakes. As a consequence, she has already been summoned to his boss.

Finally, your acquaintance stayed away from work for an entire week without an excuse. Upon her return, she seemed anxious and hounded. she reports that she is now absolutely certain, that people cannot only read other people’s thoughts, but that they also directly influence them. She was however unsure who would steer her thoughts. She also said that, when thinking, she was continually interrupted. Frequently, she would even hear those people talk to her, and they would give her instructions. Sometimes, they would also talk to each other and make fun of whatever she was doing at the time. The situation was particularly bad at her apartment, she claimed. At home, she would really feel threatened, and would be terribly scared. Hence she had not spent the night at her place for the past week, but rather she had hidden in hotel rooms and hardly dared to go out.

**Major depressive disorder**

Please imagine that you find out the following about an acquaintance with whom you are occasionally doing something in your free time:

Within the past two months, your acquaintance has changed in her nature. As opposed to previously, she is down and sad without being able to make out a tangible reason for her feeling low. She appears serious and worried. There is nothing anymore that will make her laugh. She hardly ever talks, and if she says something, she speaks in a low tone of voice about the worries she has with regard to her future. Your acquaintance feels useless and has the impression to do everything wrong. All attempts to cheer her up have failed. She lost all interest in things and is not motivated to do anything. She complains of often waking up in the middle of the night and not being able to get back to sleep. Already in the morning, she feels exhausted and without energy. She says that she encounters difficulty in concentrating on her job. In contrast with previous times, everything takes her very long. She hardly manages her workload. As a consequence, she has already been summoned to her boss.

**Supplement Table 1.** Predictors of agreement with statements about depression or schizophrenia over the last decade.

| Statement (*N*) | Predictor | Response category | Predictor estimates | | Predictor evaluation | | Model evaluation | |
| --- | --- | --- | --- | --- | --- | --- | --- | --- |
|  |  |  | β (*SE*) | Relative-risk ratio (95% *CI*) | Wald’s χ² (*df*=2) | *p* | Likelihood-ratio χ² (*df*=16) | McFadden’s Pseudo-*R*² |
| Continuum belief (5416) | Period (0=2011, 1=2020) | Agree vs. undecided | 0.22 (0.09)* | 1.24 (1.04, 1.48) | 5.57 | 0.062 | 531.44*** | 0.05 |
|  |  | Disagree vs. undecided | 0.13 (0.10) | 1.14 (0.93, 1.40) |  |  |  |  |
|  | Vignette (0=depression, 1=schizophrenia) | Agree vs. undecided | -0.20 (0.11) | 0.82 (0.67, 1.01) | 154.22 | <0.001*** |  |  |
|  |  | Disagree vs. undecided | 0.98 (0.10)*** | 2.65 (2.16, 3.26 |  |  |  |  |
|  | Period*vignette | Agree vs. undecided | -0.52 (0.14)*** | 0.60 (0.45, 0.79) | 16.18 | <0.001*** |  |  |
|  |  | Disagree vs. undecided | -0.06 (0.14) | 0.95 (0.72, 1.25) |  |  |  |  |
|  | Age of respondent | Agree vs. undecided | 0.00 (0.00) | 1.00 (1.00, 1.01) | 0.79 | 0.675 |  |  |
|  |  | Disagree vs. undecided | 0.00 (0.00) | 1.00 (1.00, 1.01) |  |  |  |  |
|  | Gender of respondent (0=female, 1=male) | Agree vs. undecided | -0.02 (0.07) | 0.98 (0.85, 1.12) | 1.99 | 0.369 |  |  |
|  |  | Disagree vs. undecided | 0.07 (0.07) | 1.07 (0.93, 1.22) |  |  |  |  |
|  | Gender of vignette (0=female, 1=male) | Agree vs. undecided | -0.01 (0.07) | 0.99 (0.86, 1.13) | 0.23 | 0.890 |  |  |
|  |  | Disagree vs. undecided | -0.03 (0.07) | 0.97 (0.85, 1.11) |  |  |  |  |
|  | Education (0=”< 10 years”, 1=”10 years”) | Agree vs. undecided | 0.19 (0.08)* | 1.20 (1.02, 1.42) | 5.96 | 0.051 |  |  |
|  |  | Disagree vs. undecided | 0.03 (0.08) | 1.03 (0.88, 1.20) |  |  |  |  |
|  | Education (0=”< 10 years”, 1=”> 10 years”) | Agree vs. undecided | 0.41 (0.10)*** | 1.51 (1.24, 1.83) | 17.99 | <0.001*** |  |  |
|  |  | Disagree vs. undecided | 0.15 (0.10) | 1.16 (0.96, 1.41) |  |  |  |  |
| Unfamiliar (5405) | Period (0=2011, 1=2020) | Agree vs. undecided | 0.09 (0.11) | 1.10 (0.89, 1.36) | 24.99 | <0.001*** | 542.54*** | 0.05 |
|  |  | Disagree vs. undecided | 0.43 (0.09)*** | 1.54 (1.28, 1.85) |  |  |  |  |
|  | Vignette (0=depression, 1=schizophrenia) | Agree vs. undecided | 0.83 (0.10)*** | 2.29 (1.86, 2.80) | 173.25 | <0.001*** |  |  |
|  |  | Disagree vs. undecided | -0.48 (0.11)*** | 0.62 (0.50, 0.76) |  |  |  |  |
|  | Period*vignette | Agree vs. undecided | -0.27 (0.14) | 0.76 (0.57, 1.01) | 12.68 | 0.002** |  |  |
|  |  | Disagree vs. undecided | -0.52 (0.15)*** | 0.60 (0.45, 0.79) |  |  |  |  |
|  | Age of respondent | Agree vs. undecided | 0.00 (0.00) | 1.00 (1.00, 1.00) | 0.16 | 0.923 |  |  |
|  |  | Disagree vs. undecided | 0.00 (0.00) | 1.00 (1.00, 1.00) |  |  |  |  |
|  | Gender of respondent (0=female, 1=male) | Agree vs. undecided | 0.13 (0.07) | 1.13 (0.99, 1.30) | 12.69 | 0.002** |  |  |
|  |  | Disagree vs. undecided | -0.11 (0.07) | 0.89 (0.78, 1.03) |  |  |  |  |
|  | Gender of vignette (0=female, 1=male) | Agree vs. undecided | 0.03 (0.07) | 1.03 (0.89, 1.18) | 0.90 | 0.639 |  |  |
|  |  | Disagree vs. undecided | 0.07 (0.07) | 1.07 (0.93, 1.23) |  |  |  |  |
|  | Education (0=”< 10 years”, 1=”10 years”) | Agree vs. undecided | -0.06 (0.08) | 0.94 (0.80, 1.10) | 0.60 | 0.741 |  |  |
|  |  | Disagree vs. undecided | -0.02 (0.08) | 0.98 (0.83, 1.15) |  |  |  |  |
|  | Education (0=”< 10 years”, 1=”> 10 years”) | Agree vs. undecided | -0.14 (0.10) | 0.87 (0.72, 1.05) | 7.36 | 0.025* |  |  |
|  |  | Disagree vs. undecided | 0.11 (0.10) | 1.12 (0.92, 1.35) |  |  |  |  |
| Incompre-hensible (5399) | Period (0=2011, 1=2020) | Agree vs. undecided | -0.18 (0.11) | 0.83 (0.67, 1.04) | 25.51 | <0.001*** | 289.36*** | 0.03 |
|  |  | Disagree vs. undecided | 0.29 (0.10)** | 1.33 (1.10, 1.61) |  |  |  |  |
|  | Vignette (0=depression, 1=schizophrenia) | Agree vs. undecided | 0.22 (0.11)* | 1.25 (1.01, 1.54) | 57.70 | <0.001*** |  |  |
|  |  | Disagree vs. undecided | -0.50 (0.10)*** | 0.61 (0.50, 0.74) |  |  |  |  |
|  | Period*vignette | Agree vs. undecided | 0.33 (0.15)* | 1.39 (1.04, 1.86) | 7.57 | 0.023* |  |  |
|  |  | Disagree vs. undecided | -0.01 (0.14) | 0.99 (0.75, 1.30) |  |  |  |  |
|  | Age of respondent | Agree vs. undecided | 0.00 (0.00) | 1.00 (1.00, 1.01) | 2.28 | 0.319 |  |  |
|  |  | Disagree vs. undecided | 0.00 (0.00) | 1.00 (1.00, 1.01) |  |  |  |  |
|  | Gender of respondent (0=female, 1=male) | Agree vs. undecided | 0.09 (0.07) | 1.10 (0.95, 1.27) | 22.50 | <0.001*** |  |  |
|  |  | Disagree vs. undecided | -0.21 (0.07)** | 0.81 (0.71, 0.93) |  |  |  |  |
|  | Gender of vignette (0=female, 1=male) | Agree vs. undecided | -0.10 (0.07) | 0.90 (0.78, 1.04) | 1.95 | 0.377 |  |  |
|  |  | Disagree vs. undecided | -0.06 (0.07) | 0.94 (0.82, 1.08) |  |  |  |  |
|  | Education (0=”< 10 years”, 1=”10 years”) | Agree vs. undecided | -0.04 (0.09) | 0.96 (0.82, 1.14) | 4.02 | 0.134 |  |  |
|  |  | Disagree vs. undecided | 0.11 (0.08) | 1.12 (0.95, 1.31) |  |  |  |  |
|  | Education (0=”< 10 years”, 1=”> 10 years”) | Agree vs. undecided | -0.03 (0.10) | 0.97 (0.79, 1.29) | 22.34 | <0.001*** |  |  |
|  |  | Disagree vs. undecided | 0.35 (0.10)*** | 1.42 (1.18, 1.72) |  |  |  |  |

Caption: Results of the multinomial logistic regression analyses with period, vignette, interaction period*vignette, and gender of the vignette, as well as age, sex, and educational attainment of the respondent as predictors. * *p*<.05, ** *p*<.01, *** *p*<.001.

**Supplementary Table 2. Sensitivity of regression estimates to the analytic procedure (linear regression estimates).**

| Statement (*N*) | Depression | | | | | | |  | | Schizophrenia | | | | | | | |  | | Model evaluation | |
| --- | --- | --- | --- | --- | --- | --- | --- | --- | --- | --- | --- | --- | --- | --- | --- | --- | --- | --- | --- | --- | --- |
|  | Estimated margin | | Estimated marginal effect | | | | |  | | Estimated margin | | | Estimated marginal effect | | | | |  | |  |  |
|  | 2011 | 2020 | Δ | [95% CI] | *z* | *p* |  | | 2011 | | 2020 | Δ | | [95% CI] | *z* | *p* |  | | *Χ²* | | *Adj.R*² |
| Continuum belief (5416) | 3.23 | 3.32 | 0.09 | 0.01, 0.18 | 2.08 | .037* |  | | 2.59 | | 2.44 | -0.15 | | -0.25, -0.06 | -3.24 | .001** |  | | 615.1*** | | 0.10 |
| Unfamiliar (5405) | 2.69 | 2.45 | -0.24 | -0.33, -0.14 | -4.87 | <.001*** |  | | 3.40 | | 3.34 | -0.06 | | -0.15, 0.03 | -1.26 | .209 |  | | 650.7*** | | 0.10 |
| Incomprehen-sible (5399) | 2.63 | 2.36 | -0.27 | -0.37, -0.18 | -5.73 | <.001*** |  | | 3.06 | | 3.00 | -0.05 | | -0.15, 0.04 | -1.11 | .267 |  | | 352.6*** | | 0.06 |

Caption: Results of the sensitivity analyses using linear regressions with period, vignette, interaction period*vignette, and gender of the vignette, as well as age, sex, and educational attainment of the respondent as predictors. All covariates were evaluated at their means and the confidence intervals estimated using bootstrapping with 10000 replications. *N* sample size*.* * *p* < .05, ** *p* < .01, *** *p* < .001.
